# Ultrasonic Studies on Molecular Interactions in Binary Mixtures of *N*-Methyl Aniline with Methyl Isobutylketone, +3-Pentanone, and +Cycloalkanones at 303.15 K

**DOI:** 10.1007/s10953-013-0003-0

**Published:** 2013-05-25

**Authors:** M. Gowrisankar, P. Venkateswarlu, K. Sivakumar, S. Sivarambabu

**Affiliations:** 1 Department of Chemistry, J.K.C. College, Guntur, 522006 India; 2 Department of Chemistry, S.V. University, Tirupathi, 517502 India; 3 Department of Chemistry, S.V. Arts U.G. & P.G. College (T.T.D’S), Tirupathi, 517502 India; 4 Research Supervisor in Chemistry, Acharya Nagarjuna University, Guntur, India

**Keywords:** Ultrasonic speed, Viscosity, Ketone, Excess volume, Intermolecular interaction, *N*-methyl aniline

## Abstract

Densities, *ρ*, viscosities, *η*, and ultrasonic sound velocities *u* of pure methyl isobutylketone, diethylketone, cyclopentanone, cyclohexanone, 2-methyl cyclohexanone and those of their binary mixtures with *N*-methyl aniline were measured at 303.15 K over the entire composition range. These experimental data have been used to calculate the excess volume (*V* ^E^), deviation in ultrasonic sound velocity (∆*u*), isentropic compressibility (*κ*_*s*_), intermolecular free length (*L*_f_), excess intermolecular free length ($L_{\text{f}}^{\text{E}}$), acoustic impedance (*Z*), excess isentropic compressibility ($\kappa_S^{\text{E}}$), deviation in viscosity (∆*η*) and excess Gibbs energy of activation of viscous flow (*G* ^*E^). The viscosity data have been correlated using three equations proposed by Grunberg and Nissan, Katti and Chaudhri, and Hind et al. The excess**/**deviations have been fitted by Redlich–Kister equation and the results are discussed in terms of molecular interactions present in these mixtures.

## Introduction

The study of thermodynamic properties of binary liquid mixtures has proved to be a useful tool in elucidating the interactions that are operating between component molecules [1]. Excess thermodynamic functions, which depend on the composition, temperature and pressure of the system, are of great importance to a chemical engineer in the design of industrial separation process and to a chemist for arriving at theories of liquid mixtures. Accurate knowledge of thermodynamic properties of organic liquid mixtures has relevance in understanding the molecular interactions between the components of the mixture.

The primary objective is to measure the speeds of sound and densities of liquid systems in order to estimate the value of isentropic compressibility, which in turn is widely used to study the molecular interactions through its excess value. The experimental data in the present investigation, namely density, ultrasonic sound velocity and viscosity, were used to compute the thermodynamic functions such as excess molar volume (*V* ^E^), deviations in ultrasonic speed (∆*u*), isentropic compressibility (*κ*_*s*_), excess isentropic compressibility ($\kappa_S^{\text{E}}$), intermolecular free length (*L*_f_), excess intermolecular free length ($L_{\text{f}}^{\text{E}}$), acoustic impedance (*Z*), deviation in viscosity (∆*η*) and excess Gibbs energy of activation of viscous flow (*G* ^*E^). In principle, the interactions between component molecules can be derived from a study of deviations from the ideal behavior of properties like molar volume, compressibility and viscosity. The sign of deviation of an excess property may be negative or positive from the ideal value depending on the type and extent of interactions between unlike molecules. In the present study, the density, ultrasonic sound velocity and viscosity of pure *N*-methyl aniline (N-MA), methyl isobutylketone (MIBK), diethylketone (DEK), cyclohexanone (CH), 2-methylcyclohexanone and their mixtures were measured at 303.15 K over the entire composition range. By using this experimental data, various thermodynamic functions were calculated. Further, the experimental viscosity data were used to test the capability of semi-empirical relations of Grunberg and Nissan [2], Katti and Chaudhri [3] and Hind et al. [4]. The present work was under taken to study the effect of sign and magnitude of excess volume, excess isentropic compressibility and deviation in viscosity when *N*-methyl aniline is mixed with various aliphatic ketones. The organic liquids used in the present study are important due to their industrial applications. *N*-methyl aniline is used as an intermediate of manufactured dyes, agrochemicals and in preparation of some organic compounds. Ketones are used as solvents, polymer precursors and in the preparation of many pharmaceuticals.

We reported in our earlier communications [5, 6] excess volumes, sound velocities and viscosities for the binary mixtures of *N*-ethyl aniline and *N*,*N*-dimethyl aniline with aromatic ketones. A survey of literature has shown that thermodynamic properties of amines with 2-propanone, 2-butanone and 2-pentanone were reported [7–9]. As far we are aware no experimental excess molar volume, ultrasonic sound velocity and viscosity data of for the present systems under investigation have been reported in the literature, Hence, the present work was undertaken to study the effect of position of the carbonyl group in a ketone molecule and chain length of ketonic molecules.

## Experimental

All the chemicals used in the present work were of Analytical Reagent (AR) grade (S.D. Fine Chemicals Ltd., India) purified as described in the literature [10]. The pure samples were attained by fractional distillation and the purity of chemicals were checked by comparing the measured densities, ultrasonic sound velocity viscosity and heat capacities, which are in good agreement with literature values [10–19] and these results are given in Table 1. The purities of the samples were further confirmed by GLC, which showed single sharp peaks. Before use, the chemicals were stored over 0.4 nm molecular sieves for about 72 h to remove water and were later degassed. The binary mixtures of *N*-methyl aniline with methylisobutylketone, diethylketone, cyclopentanone (CP), cyclohexanone and 2-methylcyclohexanone were prepared in glass bottles with air tight stoppers and adequate precautions were taken to minimize losses through evaporation. The weighing of solutions was done using an Acculab ALC-210.4 digital electronic balance with an uncertainty of ±0.0001 g. The uncertainty in solution composition, expressed in mole fraction, was found to be less than 5 × 10^−4^.

After mixing the sample, the bubble-free homogeneous sample was transferred into the U-tube of the densimeter using a syringe. The density measurements were performed with a Rudolph Research Analytical digital densimeter (DDH-2911 Model), equipped with a built- in solid-state thermostat and a resident program, at the temperature 303.15 ± 0.03 K. Typically, density precisions are ±0.00005 g·cm^−3^. Proper calibration at each temperature was achieved with doubly distilled, deionized water and with air as standards. A multi frequency ultrasonic interferometer (M-82 Model, Mittal Enterprise, New Delhi, India), operated at 2 MHz, was used to measure the ultrasonic velocities in the binary liquid mixtures at the constant temperature 303.15 K controlled by a digital constant temperature water bath. The uncertainty in the measurement of ultrasonic sound velocity is ±0.2 %. The temperature stability is maintained within ±0.02 K by circulating thermostated water around the cell with a circulating pump. In order to minimize the uncertainty of the measurement, several maxima are allowed to pass and their number, 50 in the present study, is counted. All maxima are recorded with the highest swing of the needle on the micrometer scale. The total distance *d* (cm) moved by the reflector is given by *d* = *nλ*/2, where *λ* is the wave length. The frequency, *ν*, of the crystal being accurately known (2.0 MHz), the speed of sound, *u* in m·s^−1^ is calculated by using the relation *u* = *νλ*. The working of the interferometer was tested by making measurements for pure samples of benzene, toluene, chloroform, chlorobenzene, and acetone and the measured sound velocities of these liquids were in good agreement with those reported in the literature [20]. The viscosities of the pure liquids and their binary mixtures were measured by using a modified Ubbelohde capillary type viscometer [21]. The viscometer was calibrated with pure water and the liquid was allowed to stand for about 30 min in a thermostatic water bath so that thermal fluctuations in the viscometer were minimized. The accuracy in viscosity data is ±0.005 m·Pa·s

**Table 1:** Physical properties of the pure compounds at 303.15 K

Component	Experimental, *ρ* (g·cm^−3^)	Literature	Experimental, *u* (m·s^−1^)	Literature	Experimental, *η* (mPa·s)	Literature	Experimental, *Cp* (J·mol^−1^·K^−1^)	Literature
*N*-Methyl aniline	0.98172	0.98170 [11]^a^	1551.0	1497.4 [14]	1.965	1.963 [11]^a^	206.9	207.1 [17]^a^
Methylisobutylketone	0.79609	0.79609 [13]^a^	1175.0	1170.0 [15]	0.540	0.541 [13]^a^	214.5	215.8 [19]^a^
Diethylketone	0.80930	0.80932 [13]^a^	1198.0	1197.0 [16]	0.440	0.442 [13]^a^	188.4	189.6 [19]^a^
Cyclopentanone	0.93905	0.93900 [10]	1375.0	1374.0 [16]	0.996	0.995 [18]	153.8	154.5 [19]^a^
2-Methylcyclohexanone	0.92084	0.94085 [12]^a^	1346.0	1346.0 [16]	2.225		180.2	
Cyclohexanone	0.94244	0.94246 [12]^a^	1388.0	1388.0 [16]	1.812	1.810 [18]	178.8	179.3 [19]^a^

^a^298.15 K

## Theory and Calculations

The experimental values of density *ρ*, viscosity *η* and ultrasonic velocity *u*, of pure liquids and their mixtures as function of mole fraction of *N*-methyl aniline at 303.15 K are given in Table 2. The derived parameters such as *κ*_*s*_, *L*_f_, and *Z* were calculated using the following relations

1 $$ \kappa_{s} = u^{ - 2} \rho^{ - 1} $$

2 $$ L_{\text{f}} = K/u\rho^{ 1/ 2} $$

3 $$ Z = u\rho $$

In the above equations, *ρ* is the density and *u* is the ultrasonic speed of the solutions. *K* is a temperature dependent constant [22]. The deviations in excess functions from ideality provide a relatively better tool to assess the strength of interaction between the component molecules of the binary mixtures. *V* ^E^, $\kappa_S^{\text{E}}$ ∆*η*, ∆*u* and $L_{\text{f}}^{\text{E}}$ were calculated from experimental data using the following expressions:

4 $$ V^{\text{E}} = \frac{{x_{1} M_{1} + x_{2} M_{2} }}{{\rho_{\text{m}} }} - \left\{ {\frac{{x_{1} M_{1} }}{{\rho_{1} }} + \frac{{x_{2} M_{2} }}{{\rho_{2} }}} \right\} $$

5 $$ \kappa_S^{\text{E}} = \kappa_S - \kappa_S^{\text{id}} $$

6 $$ \Updelta \eta = \eta - \left( {x_{1} \eta_{1} + x_{2} \eta_{2} } \right) $$

7 $$ \Updelta u = u - \left( {x_{1} u_{1} + x_{2} u_{2} } \right) $$

8 $$ L_{f}^{\text{E}} = L_{f} - \left( {x_{1} L_{f1} + x_{2} L_{f2} } \right) $$

In the above equations, *M*_*i*_, *η*_*i*_, *u*_*i*_ and *ρ*_*i*_ represent the molecular weight, isentropic compressibility, viscosity, ultrasonic velocity and density of component *i* and *ρ*_m_, *κ*_*S*_, *η*, and *u* the corresponding values of the mixture.

**Table 2:** Experimental/calculated values of ultrasonic sound velocity (*u*), density (*ρ*), excess ultrasonic sound velocity (∆*u*), excess molar volume (*V* ^E^), excess intermolecular free length ($L_{\text{f}}^{\text{E}}$), excess acoustic impedance (*Z* ^E^), excess isentropic compressibility ($\kappa_S^{\text{E}}$), and isentropic compressibility (*κ*_*s*_) of the binary mixtures at 303.15 K

*x*_1_	*u* (m·s^−1^)	*ρ* (g·cm^−3^)	∆*u* (m·s^−1^)	*V* ^E^ (cm^3^·mol^−1^)	$L_{\text{f}}^{\text{E}}$×10^−9^ m	*Z* ^E^ × 10^−3^/kg·m^−2^·s^−1^	$\kappa_S^{\text{E}}$/TPa^−1^	$\kappa_S^{{}}$×10^−10^/Pa^−1^	*L*_f_ × 10^−10^/m
*N-MA (1)* *+* *methylisobutylketone (2)*									
0.0000	1175.0	0.79169	0.000	0.0000	0.000	0.000	0.00	9.149	2.868
0.0901	1209.7	0.80704	0.822	−0.1001	−0.753	−0.007	−2.16	8.467	2.654
0.1406	1229.3	0.81591	1.434	−0.1700	−1.097	−0.010	−3.21	8.110	2.543
0.2065	1254.9	0.82771	2.256	−0.2648	−1.460	−0.013	−4.41	7.672	2.405
0.2759	1281.9	0.84033	3.161	−0.3558	−1.743	−0.015	−5.44	7.242	2.270
0.3652	1316.3	0.85679	3.984	−0.4490	−1.957	−0.017	−6.41	6.736	2.112
0.4295	1340.9	0.86872	4.408	−0.4891	−2.016	−0.017	−6.85	6.402	2.007
0.5026	1368.5	0.88233	4.522	−0.5040	−1.993	−0.017	−7.08	6.052	1.897
0.5728	1394.8	0.89544	4.427	−0.4876	−1.891	−0.017	−7.02	5.740	1.799
0.6305	1416.1	0.90624	4.032	−0.4519	−1.750	−0.017	−6.75	5.503	1.725
0.7029	1442.7	0.91983	3.409	−0.3806	−1.515	−0.015	−6.14	5.223	1.637
0.7791	1470.5	0.93426	2.558	−0.2847	−1.201	−0.013	−5.13	4.950	1.552
0.8509	1496.5	0.94804	1.561	−0.1827	−0.851	−0.010	−3.83	4.710	1.476
0.9209	1522.0	0.96177	0.741	−0.0865	−0.471	−0.006	−2.23	4.488	1.407
1.0000	1551.0	0.97781	0.000	0.0000	0.000	0.000	0.00	4.251	1.333
*N-MA (1)* *+* *diethyl ketone (2)*									
0.0000	1198.0	0.80482	0.000	0.0000	0.000	0.000	0.00	8.657	2.714
0.0701	1223.9	0.81772	1.154	−0.0659	−0.578	−0.002	−1.57	8.164	2.559
0.1356	1248.0	0.82977	2.133	−0.1302	−1.010	−0.004	−2.81	7.738	2.426
0.1906	1268.3	0.83986	3.018	−0.1829	−1.303	−0.004	−3.68	7.402	2.320
0.2526	1291.0	0.85118	3.832	−0.2380	−1.553	−0.005	−4.47	7.049	2.209
0.3269	1318.1	0.86461	4.704	−0.2908	−1.756	−0.005	−5.17	6.657	2.087
0.3905	1341.0	0.87597	5.153	−0.3226	−1.845	−0.005	−5.55	6.348	1.990
0.4825	1373.7	0.89215	5.377	−0.3441	−1.855	−0.005	−5.76	5.939	1.862
0.5705	1404.7	0.90733	5.313	−0.3364	−1.750	−0.005	−5.60	5.586	1.751
0.6598	1435.6	0.92241	4.690	−0.2984	−1.536	−0.004	−5.08	5.260	1.649
0.7125	1453.6	0.93117	4.087	−0.2638	−1.365	−0.004	−4.61	5.082	1.593
0.7805	1476.8	0.94234	3.283	−0.2084	−1.106	−0.004	−3.83	4.865	1.525
0.8564	1502.5	0.95468	2.190	−0.1384	−0.765	−0.003	−2.73	4.639	1.456
0.9204	1524.1	0.96500	1.198	−0.0750	−0.442	−0.002	−1.61	4.461	1.398
1.0000	1551.0	0.97781	0.000	0.0000	0.000	0.000	0.00	4.251	1.333
*N-MA (1)* *+* *cyclopentanone (2)*									
0.0000	1375.0	0.93905	0.000	0.0000	0.000	0.000	0.00	5.632	1.766
0.0682	1390.6	0.94256	3.596	−0.0313	−0.163	0.004	−0.32	5.486	1.720
0.1154	1401.6	0.94502	6.289	−0.0618	−0.272	0.007	−0.51	5.386	1.689
0.1812	1417.1	0.94829	10.208	−0.0965	−0.411	0.011	−0.71	5.251	1.646
0.2451	1431.8	0.95152	13.662	−0.1441	−0.525	0.015	−0.86	5.126	1.607
0.3024	1444.5	0.95421	16.277	−0.1732	−0.603	0.010	−0.95	5.022	1.575
0.3687	1458.3	0.95727	18.408	−0.2094	−0.662	0.021	−1.01	4.912	1.540
0.4312	1470.4	0.95992	19.508	−0.2271	−0.686	0.023	−1.04	4.818	1.511
0.5025	1483.0	0.96272	19.560	−0.2325	−0.676	0.023	−1.02	4.723	1.481
0.5894	1496.5	0.96591	17.765	−0.2270	−0.613	0.021	−0.95	4.622	1.449
0.6521	1505.2	0.96798	15.430	−0.2057	−0.539	0.018	−0.86	4.559	1.430
0.7214	1514.2	0.97011	12.233	−0.1715	−0.440	0.015	−0.74	4.495	1.409
0.7925	1522.8	0.97212	8.320	−0.1240	−0.319	0.010	−0.59	4.436	1.391
0.8504	1529.9	0.97374	5.229	−0.0879	−0.220	0.006	−0.44	4.387	1.376
1.0000	1551.0	0.97781	0.000	0.0000	0.000	0.000	0.00	4.251	1.333
*N-MA (1)* *+* *2-methylcyclohexanone (2)*									
0.0000	1346.0	0.91729	0.000	0.0000	0.000	0.000	0.00	6.017	1.886
0.0851	1361.9	0.92160	−1.545	0.0456	−1.957	−0.004	−1.35	5.850	1.834
0.1425	1372.7	0.92453	−2.512	0.0788	−2.880	−0.006	−2.20	5.740	1.800
0.2069	1384.9	0.92786	−3.514	0.1158	−3.960	−0.008	−3.11	5.619	1.762
0.2804	1399.0	0.93175	−4.482	0.1535	−4.780	−0.010	−3.90	5.483	1.719
0.3569	1414.0	0.93593	−5.164	0.1839	−5.220	−0.012	−4.43	5.343	1.675
0.4368	1430.0	0.94046	−5.544	0.2044	−5.440	−0.013	−4.68	5.199	1.630
0.5069	1444.2	0.94461	−5.714	0.2085	−5.720	−0.013	−4.73	5.075	1.591
0.5824	1459.8	0.94925	−5.592	0.2010	−5.900	−0.013	−4.57	4.943	1.550
0.6602	1476.3	0.95422	−5.041	0.1805	−6.190	−0.012	−4.28	4.808	1.507
0.7251	1490.2	0.95852	−4.445	0.1532	−5.920	−0.011	−3.78	4.698	1.473
0.7905	1504.4	0.96298	−3.652	0.1186	−5.730	−0.009	−3.26	4.588	1.438
0.8526	1518.0	0.96730	−2.783	0.0833	−4.830	−0.007	−2.47	4.486	1.407
0.9125	1531.3	0.97154	−1.762	0.0479	−3.350	−0.004	−1.56	4.389	1.376
1.0000	1551.0	0.97781	0.000	0.0000	0.000	0.000	0.00	4.251	1.333
*N-MA (1)* *+* *cyclohexanone (2)*									
0.0000	1388.0	0.93756	0.000	0.0000	0.000	0.000	0.00	5.536	1.736
0.0705	1398.5	0.93997	−0.991	0.0615	−0.529	−0.002	−0.36	5.439	1.705
0.1236	1406.4	0.94177	−1.746	0.1082	−0.798	−0.003	−0.57	5.368	1.683
0.1924	1416.7	0.94428	−2.661	0.1470	−1.024	−0.005	−0.76	5.276	1.654
0.2714	1428.7	0.94718	−3.538	0.1872	−1.208	−0.007	−0.93	5.172	1.622
0.3508	1441.0	0.95008	−4.180	0.2265	−1.352	−0.008	−1.06	5.068	1.589
0.4258	1452.8	0.95296	−4.605	0.2454	−1.446	−0.009	−1.13	4.971	1.559
0.4957	1464.1	0.95574	−4.699	0.2502	−1.457	−0.009	−1.15	4.881	1.530
0.5708	1476.5	0.95883	−4.540	0.2415	−1.421	−0.008	−1.12	4.784	1.500
0.6321	1486.9	0.96148	−4.132	0.2184	−1.347	−0.008	−1.03	4.704	1.475
0.7054	1499.4	0.96465	−3.580	0.1885	−1.217	−0.007	−0.92	4.611	1.446
0.7625	1509.4	0.96718	−2.887	0.1569	−1.033	−0.006	−0.76	4.538	1.423
0.8327	1521.7	0.97028	−2.030	0.1176	−0.791	−0.004	−0.58	4.450	1.395
0.9015	1533.8	0.97333	−1.144	0.0758	−0.510	−0.002	−0.37	4.367	1.369
1.0000	1551.0	0.97781	0.000	0.0000	0.000	0.000	0.00	4.251	1.333

The excess isentropic compressibilities ($\kappa_S^{\text{E}}$) [23] for the binary mixtures were calculated using the relations:

5 $$ \kappa_{S}^{\text{E}} = \kappa_{S} - \kappa_{S}^{\text{id}} $$

where $\kappa_S^{\text{id}}$ was calculated from the relation:

9 $$ \kappa_S^{\text{id}} = \sum\limits_{i = 1}^{2} {\phi_{i} \left({\kappa_{{Si}} + \frac{{TV_{i} \alpha_{i}^{2} }}{{C_{pi} }}}\right)} - \frac{{T\left( {\sum\limits_{i = 1}^{2} {x_{i} V_{i} }} \right)\left( {\sum\limits_{i = 1}^{2} {\phi_{i} \alpha_{i} } } \right)}}{{\sum\limits_{i = 1}^{2} {x_{i} C_{pi} } }} $$

where *ϕ*_*i*_ is the ideal state volume fraction and is defined by the relation:

10 $$ \phi_{i} = \frac{{x_{i} V_{i} }}{{\sum \limits_{i = 1}^{2}{x_{i} V_{i} } }} $$

The variation of *V* ^E^, $\kappa_S^{\text{E}}$, *∆η* and *∆u* with mole fraction were fitted to the Redlich–Kister equation [24] of the type:

11 $$ Y^{\text{E}} = x_{1} x_{2} \left\{ {a_{0} + a_{1} \left( {x_{1} - x_{2} } \right) + a_{2} \left( {x_{1} - x_{2} } \right)^{2} } \right\} $$

where *Y* ^E^ is *V* ^E^, *∆u*, $\kappa_{S}^{\text{E}}$ or *∆η*. The values of *a*_0_, *a*_1_ and *a*_2_ are the coefficients of the polynomial equation and were obtained by the method of least-squares and are given in Table 3 along with standard deviation values at 303.15 K. The standard deviations are calculated by using the equation:

12 $$ \sigma \left( {Y^{\text{E}} } \right) = \left\{ {\frac{{\sum\limits_{i = 1}^{n} {\left( {Y_{\text{obs}}^{\text{E}} - Y_{\text{cal}}^{\text{E}} } \right)^{2} } }}{n - m}} \right\}^{{{\raise0.5ex\hbox{$\scriptstyle 1$} \kern-0.1em/\kern-0.15em \lower0.25ex\hbox{$\scriptstyle 2$}}}} $$

where *n* is the total number of experimental points and *m* is the number of coefficients.The excess Gibbs energy of activation of viscous flow (*G* ^*E^) is obtained by the equation:

13 $$ G^{{*{\text{E}}}} = RT\left( {\ln \eta V - \sum {x_{i} \ln \eta_{i} V_{i} } } \right) $$

where *V*_*i*_ and *V* are the molar volumes of the component *i* and molar volume of the mixture respectively; *R* and *T* have their usual meanings. Grunberg and Nissan [2] proposed the following equation for the measurement of viscosity of liquid mixtures:

14 $$ \ln \eta = x_{1} \ln \eta_{1} + x_{2} \ln \eta_{2} + x_{1} x_{2} d $$

where *d* is a parameter proportional to the interchange energy, which reflects the non-ideality of the system. Katti and Chaudhri [3] proposed the following equation:

15 $$ \ln \eta V = x_{1} \ln \eta_{1} V_{1} + x_{2} \ln \eta_{2} V_{2} + x_{1} x_{2} \frac{{W_{\text{vis}} }}{RT} $$

where *W*_vis_ */RT* is an interaction term. Hind et al. [4] suggested an equation for the viscosity of binary liquid mixtures as:

16 $$ \eta = x_{1}^{2} \eta_{1} + x_{2}^{2} \eta_{2} + 2x_{1} x_{2} H_{12} $$

where *H*_12_ is the Hind interaction parameter.

**Table 3:** Coefficients of the Redlich–Kister equation and standard deviation values at 303.15 K

Binary mixtures	Functions	*a*_0_	*a*_1_	*a*_2_	*σ*
N-MA + cyclopentanone	*V* ^E^ cm^3^·mol^−1^	−0.938	−0.037	0.557	0.003
	∆*u* m·s^−1^	78.054	−17.532	−49.86	0.042
	$\kappa_S^{\text{E}}$ TPa^−1^	−40.807	10.036	−1.908	0.021
	∆*η* mPa·s	−0.786	0.064	0.618	0.001
N-MA + methylcyclohexanone	*V* ^E^ cm^3^·mol^−1^	0.835	0.008	−0.356	0.001
	∆*u*/m·s^−1^	−22.771	−0.927	2.748	0.050
	$\kappa_S^{\text{E}}$ TPa^−1^	−49.195	−3.018	−0.184	0.026
	∆*η* mPa·s	−0.647	0.018	0.497	0.003
N-MA + cyclohexanone	*V* ^E^ cm^3^·mol^−1^	0.976	−0.080	−0.136	0.005
	∆*u* m·s^−1^	−18.744	1.615	6.897	0.032
	$\kappa_S^{\text{E}}$ TPa^−1^	−54.630	0.817	0.047	0.030
	∆*η* mPa·s	0.107	−0.002	−0.079	0.002
N-MA + 4-methyl-2-pentanone	*V* ^E^ cm^3^·mol^−1^	−2.019	−0.002	1.181	0.001
	∆*u* m·s^−1^	18.173	0.296	−11.79	0.027
	$\kappa_S^{\text{E}}$ TPa^−1^	−283.168	−25.508	−1.960	0.030
	∆*η* mPa·s	0.148	0.001	−0.021	0.001
N-MA + 3-pentanone	*V* ^E^ cm^3^·mol^−1^	−1.380	0.001	0.504	0.001
	∆*u* m·s^−1^	21.709	−0.862	−6.918	0.044
	$\kappa_S^{\text{E}}$ TPa^−1^	−99.322	−0.838	−0.008	0.029
	∆*η* mPa·s	0.222	−0.007	−0.016	0.001

## Results and Discussion

**Fig. 1:**
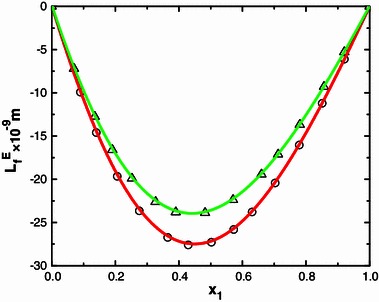
Variation of the excess intermolecular free length ($L_{\text{f}}^{\text{E}}$) with mole fraction (*x*_1_) of N-MA in the binary liquid mixtures of N-MA with methyl isobutyl ketone (*small circle*) and diethyl ketone (*small triangle*) at 303.15 K

**Fig. 2:**
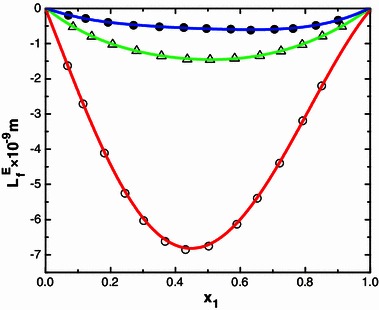
Variation of the excess intermolecular free length ($L_{\text{f}}^{\text{E}}$) with mole fraction (*x*_1_) of N-MA in the binary liquid mixtures of N-MA with cyclopentanone (*small circle*), 2-methyl cyclohexanone (*small triangle*) and cyclohexanone (*small black circle*) at 303.15 K

**Fig. 3:**
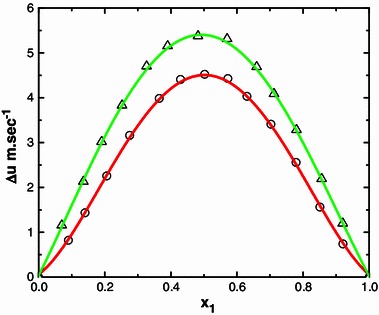
Deviation of ultrasonic speed ∆*u* with mole fraction (*x*_1_) of N-MA in the binary liquid mixtures of N-MA with methyl isobutyl ketone (*small circle*) and diethyl ketone (*small triangle*), at 303.15 K

**Fig. 4:**
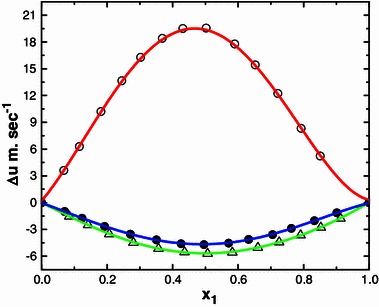
Deviation of ultrasonic speed ∆*u* with mole fraction (*x*_1_) of N-MA in the binary liquid mixtures of N-MA with cyclopentanone (*small circle*), 2-methyl cyclohexanone (*small triangle*) and cyclohexanone (*small black circle*) at 303.15 K

**Fig. 5:**
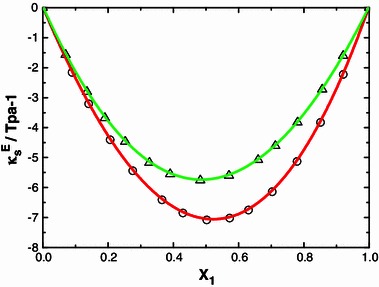
Excess of isentropic compressibility $\kappa_S^{\text{E}}$ with mole fraction (*x*_1_) of N-MA in the binary liquid mixture of N-MA with methyl isobutyl ketone (*small circle*) and diethyl ketone (*small triangle*), at 303.15 K

**Fig. 6:**
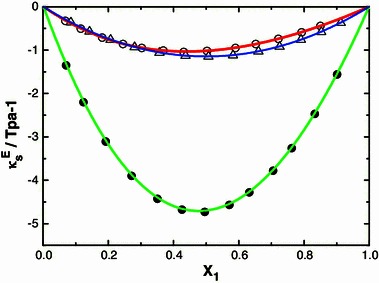
Excess isentropic compressibility $\kappa_S^{\text{E}}$ with mole fraction (*x*_1_) of N-MA in the binary liquid mixture of N-MA with cyclopentanone (*small circle*), cyclohexanone (*small triangle*) and 2-methyl cyclohexanone (*small black circle*) at 303.15 K

Ultrasound waves are high frequency mechanical waves. Their velocities in a medium depend inversely on the density and compressibility of the medium. The variation of ultrasonic velocity in a mixture depends upon the increase or decrease of intermolecular free length (*L*_*f*_) after mixing the components the computed $L_{\text{f}}^{\text{E}}$ and is graphically represented in Figs. 1, 2, 3 and 4. Further, ($\kappa_{S}^{\text{E}}$) values are calculated as described [23] are also graphically represented in Figs. 5 and 6 for binary mixtures of *N*-methylaniline with all ketones. Generally, negative values of ∆*u* indicate dispersion forces due to weak interactions whereas positive values of ∆*u* indicate strong interactions [25, 26]. The sign and magnitude of ∆*u* play important roles in describing molecular rearrangements among the component molecules in the mixtures which reflect intermolecular interactions between the molecules. A perusal of data in Table 2 shows that the values of ∆*u* are positive for N-MA + DEK, N-MA + MIBK, and N-MA + CP, whereas those for the mixtures of N-MA + CH and N-MA + Me-CH are negative over the entire composition ranges at 303.15 K.

An examination of data in Table 2 shows that the excess isentropic compressibility ($\kappa_S^{\text{E}}$) and excess intermolecular free length ($L_{\text{f}}^{\text{E}}$) are negative in all of the binary systems over the entire range of composition. According to Sri Devi et al. [27], negative excess values are due to closely packed molecules which accounts for the existence of strong molecular interactions, whereas positive excess values reflect weak interactions between unlike molecules. The sign of the excess isentropic compressibility ($\kappa_S^{\text{E}}$) and excess intermolecular free length ($L_{\text{f}}^{\text{E}}$) are useful in assessing the compaction due to molecular interactions in liquid mixtures through: hydrogen-bonding, charge transfer, dipole–dipole and dipole-induced dipole interactions, interstitial accommodation and orientational ordering [28], which lead to a more compact structure, leading to negative values of the excess isentropic compressibility and excess intermolecular free length. Hence negative values of the excess isentropic compressibility ($\kappa_S^{\text{E}}$) and excess intermolecular free length ($L_{\text{f}}^{\text{E}}$) in the present systems suggests that strong molecular interactions are present between unlike molecules in the liquid mixtures.

The $\kappa_S^{\text{E}}$ values for the systems containing methyl isobutyl ketone, diethyl ketone and alicyclic ketones fall in the orders: methyl isobutyl ketone > diethyl ketone and cyclopentanone > 2-methylcyclohexanone > cyclohexanone.

The order of alicyclic ketones is cyclopentanone > 2-methylcyclohexanone > cyclohexanone which suggests that an increase in cyclic structure hinders the interaction. The higher values for 2-methylcyclohexanone solutions compared to cyclohexanone may be due to the presence of the methyl group which increases the negative charge on the oxygen atom of the carbonyl group [16]. Hence, the above order may be justified, suggesting that dipole-dipole interactions between unlike molecules are prevailing [29].

**Fig. 7:**
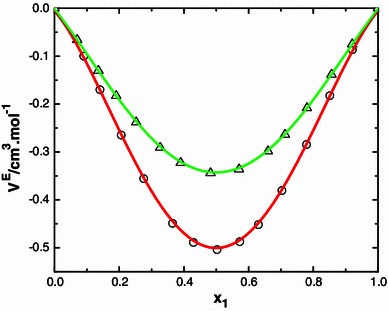
Variation of excess molar volume (*V* ^E^) with mole fraction (*x*_1_) of N-MA in the binary liquid mixtures of N-MA with methyl isobutyl ketone (*small circle*) and diethyl ketone (*small triangle*) at 303.15 K

**Fig. 8:**
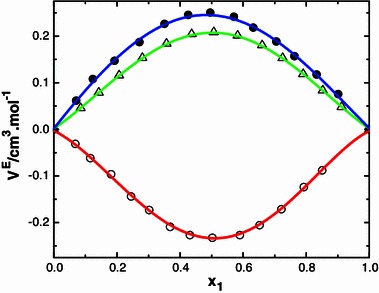
Variation of excess molar volume (*V* ^E^) with mole fraction (*x*_1_) of N-MA in the binary liquid mixtures of N-MA with cyclopentanone (*small circle*), 2-methyl cyclohexanone (*small triangle*) and cyclohexanone (*small black circle*) at 303.15 K

A careful study of data in the Table 2 suggests that the excess volume data for the systems N-MA + MIBK, N-MA + DEK, and N-MA + CP are negative whereas for the mixtures of N-MA + CH, N-MA + Me-CH are positive over the entire composition ranges at 303.15 K. The excess volume data of the binary systems of *N*-methylaniline with ketones are graphically represented in Figs. 7 and 8, it can be explained qualitatively by taking into consideration the following factors: (1) mutual loss of dipolar association due to addition of the second component and contributions due to difference in size and shape of the components, and (2) dipole–dipole and dipole-induced dipole interaction between unlike molecules, formation of H-bonds, and interstitial accommodation of the smaller molecules into voids created by the larger molecules due to the difference in molar volumes. The first factor contributes to expansion and the latter factors lead to a decrease in volume. The experimental results in the present investigation suggest that the factors responsible for contraction in volume are dominant over the entire composition range in the mixtures *N*-methyl aniline with methyl isobutyl ketone, diethyl ketone and cyclopentanone. Furthermore, the observed negative values show that there exists dipole–dipole interactions between unlike molecules and also the formation of hydrogen bonds between the oxygen atom of the carbonyl group of the ketones and the hydrogen atom of the amino group of *N*-methyl aniline. Further, *N*-methyl aniline acts as a proton acceptor and forms strong hydrogen bonds with aliphatic ketones. This hypothesis is substantiated by the considerable contraction in volume that is observed in the mixtures of *N*-methyl aniline with aliphatic ketones. The more negative *V* ^E^ values for the system methyl isobutyl ketone may be ascribed to the presence of the methyl group on the third carbon in the methyl isobutyl ketone molecule, which increases the negative charge on the oxygen atom of the carbonyl group. The negative *V* ^E^ values for the system that contains cyclopentanone may be attributed to the small ring structured cyclopentanone molecules being easily accommodated interstitially in the void space of *N*-methyl aniline. The shape and size of the ketones and their cosolvent are not similar. So, on mixing of the solvents, non-specific physical interactions and unfavorable interactions between unlike component molecules come into play thereby increasing the volume of binary solvent mixtures.

The positive *V* ^E^ values for the systems that contain cyclohexanone may be attributed to the ring structured CH molecules rupturing the hydrogen bonding in *N*-methyl aniline. Furthermore, interactions of *N*-methyl aniline with methyl cyclohexanone (Me-CH) may be ascribed to the methyl group of Me-CH.

The positive excess volume data of Me-CH is less than that of CH due to the positive inductive effect of the methyl group.

The *V* ^E^ values of N-MA with ketones follow the order: MIBK < DEK < CP < Me-CH < CH.

**Fig. 9:**
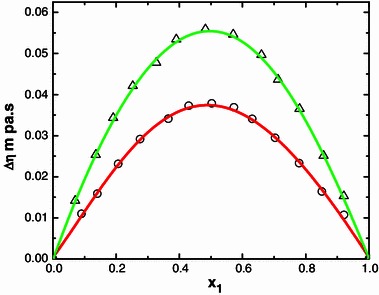
Deviation of viscosity ∆*η* with mole fraction (*x*_1_) of N-MA in the binary liquid mixtures of N-MA with methyl isobutyl ketone (*small circle*) and diethyl ketone (*small triangle*) at 303.15 K

**Fig. 10:**
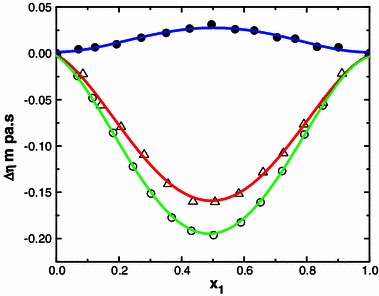
Deviation of viscosity ∆*η* with mole fraction (*x*_1_) of N-MA in the binary liquid mixtures of N-MA with cyclopentanone (*small circle*), 2-methyl cyclohexanone (*small triangle*) and cyclohexanone (*small black circle*) at 303.15 K

The deviation in viscosity (∆*η*) of all the binary mixtures are graphically represented in Figs. 9 and 10. The sign and magnitude of deviation in viscosity may depend on the combined effect of factors such as molecular size, shape and intermolecular forces [30]. In general for the systems where dispersion and dipolar interactions are operating, ∆*η* values are found to be negative [31–33], whereas charge transfer, hydrogen bonding interactions and other chemical forces lead to the formation of complex species between unlike component molecules that result in positive values of ∆*η*. The actual values depend upon the dominant factor [34]. An examination of data in the Table 4 shows that the values of ∆*η* for the systems N-MA + MIBK, N-MA + DEK and N-MA + CH are positive whereas for the mixtures of N-MA + CP and N-MA + Me-CH they are negative over the entire composition ranges at 303.15 K.

**Table 4:** Experimental and calculated values of viscosity (*η*), deviation in viscosities (∆*η*), excess Gibbs energy of activation of viscous flow (*G* ^*E^), Grunberg–Nissan interaction parameters (*d*), Katti–Chaudhri interaction parameters (*W*_vis_/*RT*), and Hind interaction parameters (*H*_12_) at 303.15 K

*x*_1_	*η* (mPa·s)	∆*η* (mPa.s)	*G* ^*E^ (J·mol^−1^)	*d*	*W*_vis_ **/** *RT*	*H*_12_
*N-MA (1)* *+* *methylisobutylketone (2)*						
0.0000	0.525	0.000	0.000			
0.0901	0.645	0.011	0.107	0.520	0.520	1.197
0.1406	0.711	0.015	0.148	0.486	0.485	1.196
0.2065	0.798	0.023	0.188	0.456	0.454	1.201
0.2759	0.888	0.029	0.213	0.426	0.424	1.203
0.3652	1.001	0.034	0.227	0.391	0.389	1.204
0.4295	1.082	0.037	0.228	0.372	0.369	1.206
0.5026	1.171	0.037	0.219	0.350	0.347	1.206
0.5728	1.255	0.036	0.203	0.332	0.329	1.205
0.6305	1.322	0.034	0.184	0.317	0.314	1.203
0.7029	1.405	0.029	0.157	0.300	0.297	1.201
0.7791	1.491	0.023	0.122	0.284	0.282	1.198
0.8509	1.571	0.016	0.086	0.270	0.269	1.195
0.9209	1.650	0.010	0.049	0.264	0.264	1.204
1.0000	1.735	0.000	0.000			
*N-MA (1)* *+* *diethylketone (2)*						
0.0000	0.425	0.000	0.000			
0.0701	0.531	0.014	0.135	0.827	0.823	1.189
0.1356	0.628	0.025	0.217	0.740	0.736	1.188
0.1906	0.709	0.034	0.265	0.686	0.681	1.191
0.2526	0.798	0.042	0.298	0.632	0.627	1.191
0.3269	0.901	0.047	0.316	0.575	0.570	1.189
0.3905	0.990	0.053	0.321	0.541	0.535	1.192
0.4825	1.113	0.055	0.307	0.494	0.489	1.192
0.5705	1.227	0.054	0.279	0.457	0.451	1.192
0.6598	1.339	0.049	0.237	0.425	0.419	1.191
0.7125	1.402	0.043	0.207	0.406	0.401	1.186
0.7805	1.484	0.036	0.165	0.387	0.382	1.187
0.8564	1.572	0.025	0.112	0.365	0.361	1.182
0.9204	1.646	0.015	0.064	0.352	0.348	1.184
1.0000	1.735	0.000	0.000			
*N-MA (1)* *+* *cyclopentanone (2)*						
0.0000	0.996	0.000	0.000			
0.0682	1.022	−0.024	−0.012	−0.082	−0.075	1.173
0.1154	1.033	−0.048	−0.028	−0.117	−0.110	1.129
0.1812	1.044	−0.086	−0.056	−0.156	−0.150	1.076
0.2451	1.055	−0.122	−0.083	−0.184	−0.178	1.035
0.3024	1.068	−0.151	−0.104	−0.201	−0.196	1.006
0.3687	1.091	−0.177	−0.121	−0.211	−0.206	0.984
0.4312	1.123	−0.192	−0.127	−0.211	−0.206	0.974
0.5025	1.171	−0.196	−0.125	−0.203	−0.198	0.972
0.5894	1.249	−0.183	−0.107	−0.180	−0.176	0.988
0.6521	1.317	−0.161	−0.087	−0.158	−0.153	1.010
0.7214	1.402	−0.127	−0.061	−0.126	−0.121	1.049
0.7925	1.494	−0.088	−0.035	−0.090	−0.085	1.099
0.8504	1.568	−0.056	−0.018	−0.062	−0.056	1.143
1.0000	1.735	0.000	0.000			
*N-MA (1)* *+* *2-methylcyclohexanone (2)*						
0.0000	2.225	0.000	0.000			
0.0851	2.161	−0.022	−0.007	−0.045	−0.040	1.836
0.1425	2.099	−0.056	−0.023	−0.081	−0.076	1.750
0.2069	2.044	−0.079	−0.034	−0.088	−0.083	1.737
0.2804	1.978	−0.109	−0.049	−0.103	−0.097	1.708
0.3569	1.909	−0.141	−0.067	−0.122	−0.116	1.672
0.4368	1.851	−0.160	−0.079	−0.133	−0.127	1.654
0.5069	1.816	−0.160	−0.080	−0.134	−0.128	1.658
0.5824	1.788	−0.151	−0.077	−0.132	−0.126	1.668
0.6602	1.773	−0.128	−0.065	−0.122	−0.116	1.693
0.7251	1.762	−0.107	−0.055	−0.115	−0.109	1.709
0.7905	1.761	−0.076	−0.038	−0.098	−0.092	1.748
0.8526	1.754	−0.053	−0.026	−0.089	−0.083	1.768
0.9125	1.756	−0.021	−0.009	−0.053	−0.047	1.843
1.0000	1.735	0.000	0.000			
*N-MA (1)* *+* *cyclohexanone(2)*						
0.0000	1.812	0.000	0.000			
0.0705	1.811	0.004	0.003	0.016	0.021	1.807
0.1236	1.809	0.006	0.005	0.014	0.019	1.803
0.1924	1.807	0.009	0.007	0.015	0.020	1.805
0.2714	1.808	0.016	0.012	0.021	0.025	1.816
0.3508	1.807	0.022	0.016	0.023	0.028	1.821
0.4258	1.806	0.026	0.019	0.027	0.031	1.828
0.4957	1.805	0.031	0.022	0.030	0.035	1.835
0.5708	1.794	0.026	0.018	0.026	0.030	1.826
0.6321	1.788	0.024	0.017	0.026	0.030	1.826
0.7054	1.775	0.017	0.013	0.020	0.025	1.815
0.7625	1.769	0.015	0.011	0.021	0.025	1.816
0.8327	1.755	0.007	0.005	0.013	0.016	1.799
0.9015	1.749	0.006	0.005	0.018	0.022	1.809
1.0000	1.735	0.000	0.000

The ∆*η* values for the systems containing methyl isobutyl ketone, diethyl ketone and alicyclic ketones fall in the order: methyl isobutyl ketone < diethyl ketone and cyclopentanone < methylcyclohexanone < cyclohexanone (Fig. 8).

According to Reed and Taylor [35] positive deviations in *G* ^*E^ may be due to specific interactions like hydrogen bonding and charge transfer, whereas the negative deviations may be ascribed to dispersion forces within the systems. An examination of data in the Table 3 suggests that the values of *G* ^*E^ for the systems N–MA + MIBK, + DEK, + CH are positive whereas for the mixtures of N-MA + CP, + Me-CH are negative over the entire composition ranges at 303.15 K. In the present investigation, positive values of *G* ^*E^ may be attributed to dipole–dipole interactions between the component molecules and the negative values show the dispersion forces. Recently, Ali et al. attributed the positive values of *G* ^*E^ in liquid mixtures to hydrogen bond formation between unlike molecules.

The interaction parameter *d*, in the Grunberg and Nissan equation, is a measure of the strength of interaction between the mixing components. Table 4 shows that the values of *d* are negative for the systems: *N*-methyl aniline + cyclopentanone and *N*-methyl aniline + methylcyclohexanone and are positive for the systems: *N*-methyl aniline + methyl isobutyl ketone, *N*-methyl aniline + diethyl ketone, and *N*-methyl aniline + cyclohexanone at 303.15 K. According to Kalra et al. [36], large and positive *d* values indicate strong specific interaction, small positive values indicate weak specific interaction and large negative values indicate no specific interaction. Hence, the negative values of *d* may be attributed to the dominance of dispersion forces arising from the breaking of hydrogen bonds in the associated component of the mixtures and positive *d* values due to specific interactions.

## Conclusions

In this paper, the densities, viscosities and speed of sound at 303.15 K have been measured over the entire range of composition of *N*-methyl aniline with ketones. From these measured physico-chemical data, excess molar volumes, deviation in viscosities, deviation in ultrasonic sound velocities and excess isentropic compressibility have been calculated. These data were correlated by a Redlich–Kister type polynomial equation to derive the coefficients and standard deviation. The results are interpreted in terms of molecular interactions between the component molecules.
